# Accidental ingestion of a dental bur in an 84‐year‐old male

**DOI:** 10.1002/ccr3.5488

**Published:** 2022-03-06

**Authors:** Francesk Mulita, Ioannis Panagiotopoulos, Georgios‐Ioannis Verras, Elias Liolis, Levan Tchabashvili, Themistoklis Paraskevas, Fotios Iliopoulos, Dimitrios Bousis, Christos Michailides, Charalampos Kaplanis, Ioannis Perdikaris, Konstantinos Bouchagier, Dimitrios Filis, Dimitrios Velissaris

**Affiliations:** ^1^ Department of General Surgery General University Hospital of Patras Patras Greece; ^2^ Department of Cardiothoracic Surgery General University Hospital of Patras Patras Greece; ^3^ Department of Internal Medicine General University Hospital of Patras Patras Greece; ^4^ Department of Surgery "St. Andrew" General Hospital of Patras Patras Greece

**Keywords:** dental bur, endoscopy, foreign body, X‐rays

## Abstract

This report describes the case of an 84‐year‐old male who was brought to the emergency room because a dental bur was swallowed accidentally during a dental procedure. The foreign body was successfully removed by gastroenterologists endoscopically 8 days after the ingestion and was identified as a 2‐cm‐long dental bur.

## Graphical abstract

Although the majority of ingested foreign bodies pass through the gastrointestinal tract, endoscopic intervention is necessary in 10%–20% of these cases.

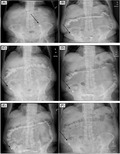

## 1 INTRODUCTION

Ingestion of foreign bodies is very common. Especially, more than 120,000 cases were recorded in the United States in only 1 year, while approximately 1500 deaths per year are attributed to ingestion of foreign bodies.^1^, ^2^ Various complications can be occurred during daily clinical practice by accidental ingestion of dental objects such as burs, impression materials, dental inlays or crowns, endodontic posts, and fixed or removable prosthetic restorations.^3^ These life‐threatening complications like peritonitis, sepsis, fistulas and duodenocolic fissures, abscess formation, and injury to the digestive tract are related to obstruction or perforation caused by the ingested foreign body.^1^, ^3^ Ingested foreign bodies usually pass through the anus without any complication. In 10%–20% of patients, endoscopic removal is required, while in 1% of patients surgery is necessary.^3^ We herein report a case of an accidental ingestion of a dental bur in an 84‐year‐old male.

## 2 CASE REPORT

**FIGURE 1:**
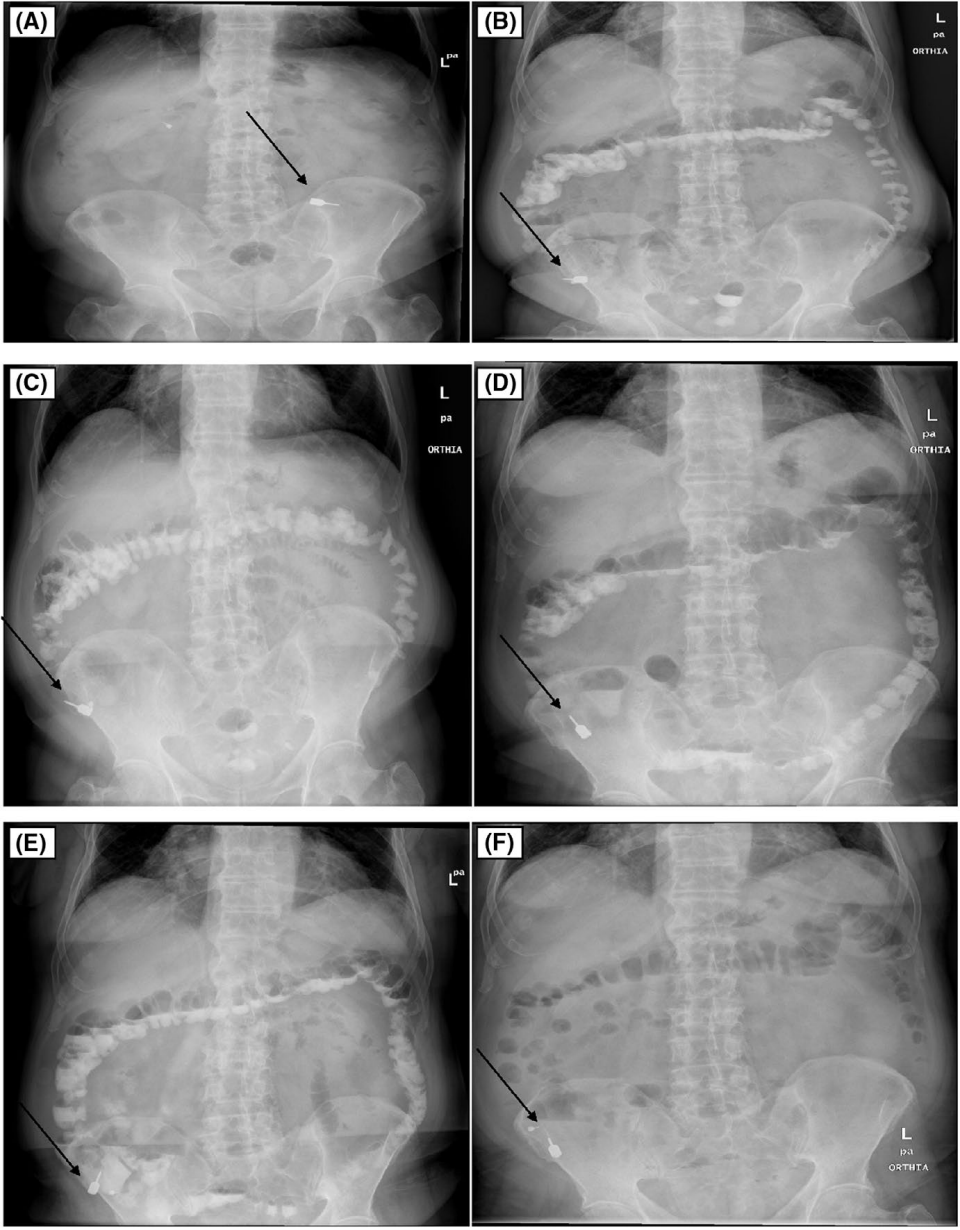
Abdominal radiography: showed a hyperdense, foreign body in the left lower quadrant 2 hours after ingestion (A). Movement of foreign body in the right lower quadrant 24 hours after ingestion (B). The foreign body remains in the same position 48 hours (C), 72 hours (D), 5 days (E), and 7 days (F) after ingestion

An 84‐year‐old male with atrial fibrillation, hypertension, and type 2 diabetes mellitus was brought to the emergency room of our hospital by a private dental practitioner. The dentist disclosed that a 2‐cm dental bur was swallowed accidentally by his patient 2 hours ago during the dental procedure. On examination, the patient's temperature was 36.5, heart rate was 71 beats per minute, blood pressure was 151/87, and respiratory rate was 17 breaths per minute. His abdomen was soft, without distension and with no evidence of palpable mass. His routine blood tests including hemogram, C‐reactive protein level, liver and renal function test, serum amylase, and lipase were normal. Chest radiography showed no evidence of free air and no other abnormalities. X‐ray of the abdomen revealed a hyperdense, foreign body in the left lower quadrant (Figure 1A). Upper endoscopy was not performed as the foreign body was shown distal to stomach—not accessible to endoscopy.

Patient was kept under close observation in the department of surgery. He was kept under high fibrous diet, and 20 ml of oral gastrografin was prescribed every 8 hours for allowing the rapid transport of the foreign object within the gastrointestinal (GI) tract. Serial radiographic examinations were performed to monitor the migration of the ingested foreign body. A repeated x‐ray of the abdomen was taken 24 hours after the ingestion of the object. This radiograph suggested the presence of the foreign body in the right lower quadrant of the abdomen (Figure 1B). As the patient did not agree to make any endoscopic or surgical intervention, abdominal radiographies were performed 48 hours (Figure 1C), 72 hours (Figure 1D), 5 days (Figure 1E), and 7 days (Figure 1F) after the foreign body ingestion, but the object remained in the same position.

An attempt was made by gastroenterologists to remove the foreign body endoscopically. Eventually, anterograde colon preparation and colonoscopy were performed. The foreign body was detected in terminal ileum, was grasped with an endoscopic loop, pulled through the valve, and removed per anus. The length of the dental bur was about 2 cm. The patient did not have any complication, and he was mobilized 2 hours after the foreign body removal. He was started on oral diet 12 hours afterward and was discharged home in a very good condition 24 hours after the endoscopy.

## 3 DISCUSSION

Foreign bodies may be ingested, inserted into a body cavity, or deposited into the body by a traumatic or iatrogenic injury. The presentation of foreign body in the emergency department varies greatly and can suffer from incorrect or delayed diagnosis. Factors affecting the acuity of the problem include the object that is ingested, the location of the object, whether the event was witnessed, the age of the patient, as well as the timeframe in which ingestion occurred.^4^, ^5^ Ingestion of foreign body is commonly encountered in radiological practice, and its significance should not be underestimated.^1^ Whenever a dentist loses a dental material or any other foreign object inside oral cavity, a radiologist must be consulted even if the patient has no symptoms.^6^ Usually, the foreign bodies pass through the GI tract and are evacuated in 2–5 days without symptoms.^3^ The two most common locations of an ingested foreign object at the initial presentation are in the stomach (58.1%) and small intestine (32.7%).^7^ Endodontic files have been reported to pass through GI tract within 3 days, while 10%–20% require endoscopic intervention and only in 1% surgery is necessary.^8^

Radiographic examination with chest and abdominal x‐ray is necessary for determining the location, size, and nature of the ingested foreign object.^9^ The computed tomography (CT) scan can also be used to localize foreign objects, but it is usually performed to define the exact extent of injury to the involved organs and the damage to the surrounding tissues.^1^ In our case, the foreign body was radio opaque and the size assessment was noted. We did not perform a CT scan as the foreign body was identified in radiography and the patient had normal blood tests and no symptoms.

Size, sharpness, and shape of the ingested foreign body are some characteristics that should be taken into consideration when a medical professional has to deal with this condition. The risk of injury increases when the size of the object is more than 5 cm or has a pointed shape.^10^

According to the literature, the removal of a foreign object that has entered the gastrointestinal tract is determined on the basis of patients’ age, size, shape and location of the object, and time since the ingestion. The risk of complications such as obstruction or perforation determines the timing of endoscopy.^11^, ^12^ The overall rate of perforation caused by ingested foreign bodies is in the range of 1%–7%. However, the incidence is increased to 15%–35%, when pointed or sharp foreign bodies are considered.^13^ According to Bondarde et al., the management of sharp bodies such as endodontic file, when lodged in GI tract, is endoscopic retrieval or the careful monitoring with periodic radiographs. If the foreign body does not succeed to progress after 72 hours or complications such as perforation, obstruction, or bleeding are noticed, surgery should be preferred immediately.^6^ In our case, periodic abdominal radiographies were performed for 7 days, because the patient did not agree to make any endoscopic or surgical intervention the first 7 days. Oral gastrografin was prescribed for allowing the rapid transport of the foreign body within the GI tract. The barium sulfate was not preferred to be used because of its side effects such as constipation and mechanical obstruction of bowel, which can make it difficult to perform endoscopy.^14^ In addition, laxative treatment should be given to patients after barium studies to reduce the incidence of colonic retention of barium sulfate.^15^ Fortunately, the foreign body which was identified as a 2‐cm‐long dental bur was successfully removed by gastroenterologists endoscopically 8 days after the ingestion.

## 4 CONCLUSION

Iatrogenic accidents during routine dental procedures are common and unpredictable. Early recognition and diagnosis of this condition is the key to prevent serious complications. In 10%–20% of these cases, endoscopic intervention is necessary and less than 1% of patients require surgical retrieval. In our case, the foreign body was removed by gastroenterologists endoscopically.

## CONFLICT OF INTEREST

There are no conflict of interest to declare.

## AUTHOR CONTRIBUTIONS

FM, G‐IV, EL, LT, IP, TP, CM, DB, FI, IP, and CK contributed to the clinical data collection and prepared the case report. FM, DF, KB, and DV contributed to the design of the case report presentation and performed the final revision of the manuscript.

## CONSENT

A written informed consent was obtained from the patient for publication of this case report.

## Data Availability

Data available on request from the authors.

## References

[1] Guelfguat M , DiPoce J , DiPoce J . A dental nightmare, resolved: what a radiologist needs to know when consulted about ingestion of dental foreign body material. BJR Case Rep. 2016;2:20150166. 30363664 10.1259/bjrcr.20150166 PMC6180876

[2] Amarlal D , Jeevarathan J , Muthu MS , Venkatachalapathy A , Rathna PV . Iatrogenic accidental ingestion of a dental bur. Indian J Pediatr. 2009;76(3):333‐334. doi:10.1007/s12098-009-0003-7 19205636

[3] Dionysopoulos D . Accidental ingestion and aspiration of foreign objects during dental practice. Stomatological Dis Sci. 2017;1:87‐89.

[4] Mulita F , Theofanis G , Tchabashvili L , Drakos N , Maroulis I . Rectal foreign bodies: retained orange. Prz Gastroenterol. 2021;16(4):392‐393. doi:10.5114/pg.2021.105409 34976250 PMC8690951

[5] Mulita F , Troupi IM , Liolis E , Tchabashvili L , Perdikaris I , Maroulis I . Rectal foreign bodies: the role of gender. Seksuologia Polska. 2021;19. doi:10.5603/SP.2021.0013

[6] Bondarde P , Naik A , Patil S , Shah PH . Accidental ingestion and uneventful retrieval of an endodontic file in a 4 year old child: a case report. J Int Oral Health. 2015;7(Suppl 2):74‐76. PMC4672839 26668487

[7] Antoniou D , Christopoulos‐Geroulanos G . Management of foreign body ingestion and food bolus impaction in children: a retrospective analysis of 675 cases. Turk J Pediatr. 2011;53:381‐387. 21980840

[8] Bhatt R , Atrey A , Kaur A , Dave L . Accidental ingestion of a dental bur seen in a paediatric patient ‐ a case report. Adv Hum Biol. 2013;3(3):82‐84.

[9] Daneswari V , Visalaxi HR . Emergency management of an accidental ingestion of a dental foreign body in pediatric patient using rigid esophagoscopy ‐ a case report. Pediatr Dent Care. 2016;1:111. doi:10.4172/pdc.1000111

[10] Jaan A , Mulita F . Gastrointestinal foreign body. In: StatPearls [Internet]. StatPearls Publishing; 2020. PMID: 32965874. 32965874

[11] Mulita F , Papadopoulos G , Tsochatzis S , Kehagias I . Laparoscopic removal of an ingested fish bone from the head of the pancreas: case report and review of literature. Pan Afr Med J. 2020;36:123. doi:10.11604/pamj.2020.36.123.23948. Published 2020 Jun 25. 32849978 PMC7422735

[12] Mulita F , Kehagias D , Tchabashvili L , Iliopoulos F , Drakos N , Kehagias I . Laparoscopic removal of a fishbone migrating from the gastrointestinal tract to the pancreas. Clin Case Rep. 2021;9(3):1833‐1834. doi:10.1002/ccr3.3822 33768960 PMC7981611

[13] Brinster CJ , Singhal S , Lee L , Marshall MB , Kaiser LR , Kucharczuk JC . Evolving options in the management of esophageal perforation. Ann Thorac Surg. 2004;77(4):1475‐1483. doi:10.1016/j.athoracsur.2003.08.037 15063302

[14] Khartade HK , Meshram VP , Tumram NK , Parchake MB , Pathak HM . Fatal aspiration of barium sulfate in a case of myasthenia gravis: a case report and review of literature. J Forensic Sci. 2020;65(4):1350‐1353. doi:10.1111/1556-4029.14297 32069365

[15] Pathan S , Benzar T , Master S , Peddi P . Iatrogenic constipation from barium blockade: a case report. Clin Case Rep. 2019;7(8):1562‐1564. Published 2019 Jul 10. doi:10.1002/ccr3.2280 31428392 PMC6692969

